# Prediction of sgRNA on-target activity in bacteria by deep learning

**DOI:** 10.1186/s12859-019-3151-4

**Published:** 2019-10-24

**Authors:** Lei Wang, Juhua Zhang

**Affiliations:** 1 School of Life Science, Beijing Institute of Technology, South Zhongguancun Street, Beijing, 100081 China; 2 Key Laboratory of Convergence Medical Engineering System and Healthcare Technology, The Ministry of Industry and Information Technology, Beijing Institute of Technology, Beijing, China

**Keywords:** CRISPR-Cas9, On-target activity, Prokaryotes, Deep learning

## Abstract

**Background:**

One of the main challenges for the CRISPR-Cas9 system is selecting optimal single-guide RNAs (sgRNAs). Recently, deep learning has enhanced sgRNA prediction in eukaryotes. However, the prokaryotic chromatin structure is different from eukaryotes, so models trained on eukaryotes may not apply to prokaryotes.

**Results:**

We designed and implemented a convolutional neural network to predict sgRNA activity in *Escherichia coli*. The network was trained and tested on the recently-released sgRNA activity dataset. Our convolutional neural network achieved excellent performance, yielding average Spearman correlation coefficients of 0.5817, 0.7105, and 0.3602, respectively for Cas9, eSpCas9 and Cas9 with a *recA* coding region deletion. We confirmed that the sgRNA prediction models trained on prokaryotes do not apply to eukaryotes and vice versa. We adopted perturbation-based approaches to analyze distinct biological patterns between prokaryotic and eukaryotic editing. Then, we improved the predictive performance of the prokaryotic Cas9 system by transfer learning. Finally, we determined that potential off-target scores accumulated on a genome-wide scale affect on-target activity, which could slightly improve on-target predictive performance.

**Conclusions:**

We developed convolutional neural networks to predict sgRNA activity for wild type and mutant Cas9 in prokaryotes. Our results show that the prediction accuracy of our method is improved over state-of-the-art models.

## Background

Gene editing allows modification of the genome and transcription products on target sites. The CRISPR-Cas9 system is a bacterial adaptive immune system, which includes CRISPR-associated nuclease Cas9 (SpCas9), a specificity-determining CRISPR RNA (crRNA), and an auxiliary trans-activating RNA (tracrRNA) [1–3]. The crRNA and tracrRNA duplexes can be fused to generate a chimeric single-guide RNA (sgRNA), which targets the complex to a 3’NGG-flanked genomic region [4–6] protospacer adjacent motif (PAM) via ∼20 nucleotide Watson-Crick base pairing [7]. During DNA double-stranded break (DSB) induction and subsequent nonhomologous end joining (NHEJ) DNA damage repair, specific genomic fragments can be inserted, deleted or replaced. Therefore, the system can be reprogrammed by changing the sgRNA sequence for site-specific editing [2, 8, 9], allowing investigation of gene function [10, 11], gene expression [12, 13], genetic interactions [14, 15], and the relationships between genetic variations and phenotypes [16, 17]. Moreover, CRISPR-Cas9 has been applied to clinical trials, editing and remodeling harmful genes for personalized therapy [18, 19].

CRISPR-Cas9 using a specific sgRNA can precisely edit the target site (i.e. on-target editing), though it may bind and edit at other additional sites (i.e. off-target editing). Off-target effects are undesired and should be minimized. Moreover, widely varying sgRNA on-target activity limits further application of CRISPR-Cas9 gene editing [20–22]. Poor sgRNA activity results in a high false-positive rate during genome editing, which allows many wild-type cells to survive [23]. Thus, designing criteria to maximize sgRNA efficacy is necessary to improve success and reproducibility. Various sgRNA design rules and tools have been developed for sgRNA on-target efficacy classification and regression in eukaryotes. Some learning-based methods have achieved better performance, such as sgRNA Designer [22], SSC [24], sgRNA Score [25, 26], CRISPRscan [27], TSAM [28] and DeepCRISPR [29].

Some studies indicate that the CRISPR-Cas9 system is affected by chromatin structures in eukaryotic cells. Chromatin openness and CRISPR-Cas9 mutagenesis efficiency are correlated, indicating that CRISPR-Cas9 mutagenesis is influenced by chromatin accessibility in zebrafish embryos [30]. Mapping genome-wide binding sites of a catalytically-inactive Cas9 (dCas9) in mouse embryonic stem cells (mESCs) demonstrated that chromatin inaccessibility prevents dCas9 binding to target sites [31]. When the DNA target is within a nucleosome, strong Cas9 cleavage inhibition occurs in yeast cells, which is relieved when nucleosomes are depleted [32, 33]. The prokaryotic genome is occupied by nucleoid-associated proteins [34] and transcription factor binding [35], but lacks complex chromatin structures [36]. However, there are inactive sgRNAs during genome editing in prokaryotic cells [37–40], so optimizing sgRNA activity is also necessary for prokaryotes. Meanwhile, sgRNA activity prediction models trained on eukaryotes do not apply to prokaryotes [23, 40]. Guo et al. [23] found a very weak correlation between prokaryotic datasets and predictions from two eukaryotic machine learning models (Doench et al. [22] and Xu et al. [24]) and a notable but weak negative correlation with biophysical model predictions (Farasat et al. [41]). Cui and Bikard used the model from Doench et al. [21] and observed very poor predictions for the activity of 13 targets in *E. coli* [40]. A gradient-boosting regression tree (GBR) has been used to predict sgRNA activity for prokaryotes [23]. Although the GBR model was predictive, modest Spearman correlation coefficients of 0.542, 0.682 and 0.328 for Cas9, eSpCas9 and Cas9, respectively (△*recA*) [23], indicates a large space for performance improvement.

Recently, a deep-learning framework, DeepCRISPR [29], was presented to predict on-target knockout efficacy and whole-genome off-target cleavage with better performance than available state-of-the-art tools. Moreover, Kim et al. [42] and Xue et al. [43] used a deep-learning framework based on one convolution layer, DeepCas9, to predict sgRNA activity in human cells. Lin and Wong designed deep convolutional and deep feedforward neural networks to predict off-target mutations for eukaryotic CRISPR-Cas9 gene editing, simultaneously demonstrating improvements over available state-of-the-art off-target prediction methods and traditional machine learning models including random forest, gradient boosting tree, and logistic regression [44]. However, sgRNA activity prediction models trained on eukaryotes are almost invalid for prokaryotes.

In this study, we developed a convolutional neural network with five convolution layers to predict sgRNA activity in prokaryotes. We created a sgRNA activity predictor for wild type and mutant Cas9 in prokaryotes, surpassing available state-of-the-art models. We confirmed that sgRNA activity prediction models trained on prokaryotes are not appropriate for eukaryotes. Then, we trained our convolutional neural network with eukaryotic data, similarly surpassing available state-of-the-art eukaryotic models. We next adopted perturbation-based approaches to analyze biological patterns between prokaryotic and eukaryotic editing. Then, we improved predictive performance of prokaryotic Cas9 by transfer learning. Finally, we observed that genome-wide potential off-target effects influence on-target activity, and utilized genome-wide accumulative potential off-target scores and sgRNA guide sequence fold scores to further improve predictive performance.

## Results

### Comparison and selection of models

We used a bacterial dataset (Set 1) with good signal-to-noise ratio and low bias, including Cas9, eSpCas9, and Cas9 (△*recA*). We removed redundancy in Set 1 with similarity threshold 0.8, which established another dataset (Set 2, see Table 1). To evaluate the performance of our models, we compared the predictive results with several other hot spot prediction methods [29, 42, 44] based on other network architectures.

**Table 1:** Number of samples and range of on-target activity value in Set 1 and Set 2

Descriptions	Cas9		eSpCas9		Cas9 (△*recA*)	
	Set 1	Set 2	Set 1	Set 2	Set 1	Set 2
Size	44,163	40,605	45,071	41,426	48,112	43,950
Min	0.0016	0.0016	0.0007	0.0007	0.0080	0.0080
Max	48.3807	48.3807	45.1725	45.1725	22.0268	22.0268
Mean	24.6415	24.6381	16.9593	16.9825	12.4479	12.4518

**Fig. 1:**
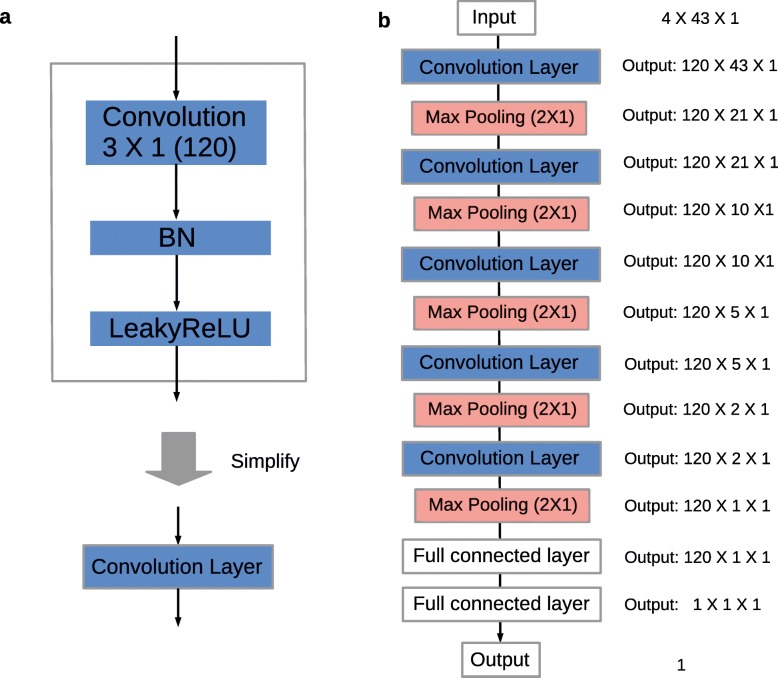
Components of constructed convolution layers and overall CNN_5layers construction. **a** The picture on the top shows details of the constructed convolution layer, which contains a convolution operation, a batch normalization and a leaky rectified linear unit in turn. The convolution kernel size is 3 ×1 and the output channel is 120. The simplified diagram is on the bottom. **b** The picture shows the overall CNN_5layers schema, including five convolution layers, five maximum pooling layers, and two fully-connected layers. All activation functions in CNN_5layers were LeakyReLU. One dropout layer which drops 30 percent was performed after each pooling operation and the first fully-connected layer

We used 5-fold cross-validation to select and compare these architectures (Table 2, and more detailed tables in Additional file 1: Table S1). Our CNN_5layers (see Methods and Fig. 1) improve prediction accuracy over others, which achieved average Spearman correlation coefficients 0.5817 (0.5787), 0.7105 (0.7063), and 0.3602 (0.3577) for Cas9, eSpCas9, and Cas9 (△*recA*), respectively, in Set 1 (Table 2), under 5-fold cross-validation. Compared with Table 2, Additional file 2: Figure S1 contains more information and demonstrates the reliability of the results and significant increases in a more visual way. We used a Steiger test for statistical significance testing between DeepCas9 (next-best model) and CNN_5layers. The *p*-values were 2.4e -12, 5.8e -7 and 5.7e -4, for Cas9, eSpCas9, and Cas9 (△*recA*), respectively, in Set 1. The *p*-values in Set 2 were similar to those in Set 1. We found that the performance of simple CNN_2layers (see Methods and Additional file 3), a CNN architecture with two convolution layers, improved prediction accuracy than those of traditional machine learning algorithms for Set 1 (Gradient Boosting Regression tree: 0.542, 0.682 and 0.328, Additional file 1: Table S1). However, DeepCRISPR [29] and CNN_Lin [44] perform poorly because of over- and under-fitting when comparing the training and test loss curves (data not shown). By comparing performance between CNN_Lin [44] and DeepCas9 [42], which have similar network architectures (only one multi-scale convolution layer) and different input size (23 nt and 30 nt respectively), we concluded that target DNA flanking sequences affect sgRNA on-target activity. Our CNN_5layers is more robust, which could largely prevent over-fitting for Cas9 and eSpCas9 (Additional file 2: Figure S1), possibly due to composite application of batch normalization and dropout. Thus, our CNN_5layers network had improved predictive performance. The performance in Set 2 were slightly weak, due to the smaller training sample size. We therefore used Set 1 for the following research.

**Table 2:** Average Spearman correlation coefficients under 5-fold cross-validation for several network architectures

Networks	Cas9		eSpCas9		Cas9 (△*recA*)	
	Set 1	Set 2	Set 1	Set 2	Set 1	Set 2
DeepCRISPR	0.5149	0.5139	0.6617	0.6631	0.3108	0.3049
CNN_Lin	0.5217	0.5214	0.6665	0.6685	0.3176	0.3144
DeepCas9	0.5554	0.5517	0.6951	0.6881	0.3400	0.3362
CNN_5layers	0.5817	0.5787	0.7105	0.7063	0.3602	0.3577

### Invalidity cross domains

Eukaryotic sgRNA activity prediction models are almost invalid for prokaryotes [23, 40]. We used eleven independent eukaryotic datasets to study the validity of our prokaryotic-trained models (Additional file 1: Table S2). Only predictions from the eSpCas9 model were statistically significant (*p*-value <0.05). All Spearman correlation coefficients are less than 0.11. Among prokaryotes, we used the eSpCas9 model to predict Cas9 activity, with a Spearman correlation coefficient of 0.5822. These results from these two different results demonstrate our model is valid within domains, but not valid between domains.

### CNN_5layers also better in eukaryotes

We trained CNN_5layers with eukaryotic data, which produced a eukaryotic model. Then, we compared our eukaryotic model with other models including DeepCRISPR [29], DeepCas9 [43] and TSAM [28]. To ensure valid comparisons, overlapping test samples relative to respective training sets were removed from eleven independent eukaryotic test sets (see Methods and Additional file 4). Similar to the prokaryotic models, DeepCRISPR [29] performed poorly (Additional file 1: Table S2). However, for nine of eleven test sets, our eukaryotic CNN_5layers model outperformed other models (Table 3). In short, our CNN_5layers network can be generalized to other eukaryotic species.

**Table 3:** Comparison of Spearman correlation coefficients between eukaryotic sgRNA activity and eukaryotic model predictions

Independent test datasets	Size	DeepCas9	TSAM_U6	CNN_5layers
chari2015Train293T	1234	—	0.3812	0.3607
doench2014HsA375	1276	0.3237	0.3187	0.3369
doench2016	2333	0.3527	0.3439	0.3945
hart2016-GbmAvg	4272	0.3795	0.4242	0.4404
hart2016-Hct1162lib1Avg	4239	0.3679	0.4161	0.4288
hart2016-Hct1162lib2Avg	3617	0.3196	0.3598	0.3829
hart2016-HelaLib1Avg	4256	0.3403	0.3879	0.4033
hart2016-HelaLib2Avg	3845	0.3617	0.3942	0.4390
hart2016-Rpe1Avg	4214	0.2519	0.3094	0.3044
wang2015hg19	2921	0.2030	0.1882	0.2291
xu2015TrainMEsc	981	0.3668	0.4088	0.4111

The DeepCas9 training set contains all chari2015Train293T samples

### Analyzing melting temperatures and RNA fold scores

We next calculated target sequence melting temperatures T(1_7), T(8_15), T(16_20), T(1_20), T(-5_-1) and T(21_+2) (see Methods). The Spearman correlation coefficients between on-target activity and melting temperatures (Table 4) are all statistically significant (*p*-values ∈(3e -203, 0.05), Additional file 1: Table S3), except for the feature T(1_7) in Cas9 (△*recA*) scenario. The melting temperatures are listed in Additional file 5. We found that T(1_20) is the most important feature, consistent with previous results utilizing relative feature importance (Gini importance) [23]. However, we observed that the second most important feature is T(16_20) for Cas9 and T(8_15) for eSpCas9. This result is also consistent with previous study results [23]. We used the six melting temperatures (combination t in Table 5) to predict on-target activity by simple linear regression. The average Spearman correlation coefficient between predictive value and on-target activity is 0.1777 (0.1604) for Cas9 (eSpCas9) in Table 6. The Spearman correlation coefficient of combination t is significantly different than feature t1, t2, t3, t4, t5, or t6.

**Table 4:** The Spearman correlation coefficients between on-target activity and six melting temperatures, four RNA fold scores, and four POSs

Abbreviations	Features	Cas9	eSpCas9	Cas9 (△*recA*)
t1	T(1_7)	-0.0302	-0.0322	—
t2	T(8_15)	-0.0915	-0.1304	-0.0579
t3	T(16_20)	-0.1424	-0.0695	-0.0789
t4	T(1_20)	-0.1439	-0.1346	-0.0762
t5	T(-5_-1)	0.0239	0.0385	0.0332
t6	T(21_+2)	0.0098	0.0107	0.0119
f1	MFE	0.0944	0.0895	0.0601
f2	FETE	0.0862	0.0832	0.0517
f3	FMSE	-0.0246	-0.0276	-0.0323
f4	ED	0.0166	0.0225	0.0286
p1	Cropit_POS	-0.1083	-0.0998	-0.0527
p2	Cctop_POS	-0.1088	-0.1003	-0.0518
p3	Mit_POS	-0.1130	-0.1073	-0.0607
p4	Cfd_POS	-0.1131	-0.0985	-0.0579

T(1_7) in Cas9 (△*recA*) scenario is not statistically significant

**Table 5:** Descriptions of several feature combinations

Combinations	Descriptions
t	t1, t2, t3, t4, t5, t6
t_c	t1, t2, t3, t4, t5, t6, c
t_p	t1, t2, t3, t4, t5, t6, p1, p2, p3, p4
t_p_c	t1, t2, t3, t4, t5, t6, p1, p2, p3, p4, c
t_p_f	t1, t2, t3, t4, t5, t6, p1, p2, p3, p4, f1, f2, f3, f4
t_p_f_c	t1, t2, t3, t4, t5, t6, p1, p2, p3, p4, f1, f2, f3, f4, c
t_f	t1, t2, t3, t4, t5, t6, f1, f2, f3, f4
t_f_c	t1, t2, t3, t4, t5, t6, f1, f2, f3, f4, c
p	p1, p2, p3, p4
p_c	p1, p2, p3, p4, c
p_f	p1, p2, p3, p4, f1, f2, f3, f4
p_f_c	p1, p2, p3, p4, f1, f2, f3, f4, c
f	f1, f2, f3, f4
c	CNN_5layers output

**Table 6:** Average performances in training set and test set under 5-fold cross-validation for fifteen feature combinations by Linear Regression

Combinations	Cas9		eSpCas9		Cas9 (△*recA*)	
	training set	test set	training set	test set	training set	test set
t	0.1782	0.1777	0.1604	0.1604	0.1070	0.1056
p	0.1217	0.1207	0.1121	0.1121	0.0630	0.0604
f	0.0962	0.0956	0.0947	0.0940	0.0674	0.0667
t_p	0.1931	0.1917	0.1772	0.1759	0.1157	0.1130
t_f	0.1888	0.1880	0.1740	0.1724	0.1191	0.1174
p_f	0.1480	0.1467	0.1408	0.1399	0.0864	0.0837
t_p_f	0.2026	0.2010	0.1892	0.1875	0.1258	0.1228
c	0.6631	0.5817	0.8060	0.7105	0.4765	0.3602
t_c	0.6631	0.5813	0.8064	0.7112	0.4747	0.3574
p_c	0.6636	0.5827	0.8068	0.7122	0.4768	0.3601
f_c	0.6650	0.5851	0.8077	0.7137	0.4773	0.3639
t_p_c	0.6637	0.5824	0.8072	0.7125	0.4753	0.3579
t_f_c	0.6655	0.5848	0.8085	0.7142	0.4753	0.3619
p_f_c	0.6656	0.5861	0.8085	0.7149	0.4775	0.3640
t_p_f_c	0.6663	0.5860	0.8092	0.7155	0.4758	0.3624

In addition, we used four physicochemical properties: minimum free energy (MFE), free energy of the thermodynamic ensemble (FETE), frequency of the minimum free energy structure in the ensemble (FMSE), and ensemble diversity (ED) to characterize the secondary structure of 20 nt-long guide RNAs using ViennaRNA [45] (see Methods). Among the four physicochemical properties, MFE is the most characteristic property (Table 4). We also used four RNA fold scores as features (combination f in Table 5) to predict on-target activity by simple linear regression. We found that the average Spearman correlation coefficient between the prediction and the true on-target activity is 0.0956 (0.0940) for Cas9 (eSpCas9) in Table 6, which achieves higher correlation coefficients than feature f1, f2, f3, and f4.

### Interpreting the learned model and transfer learning

**Fig. 2:**
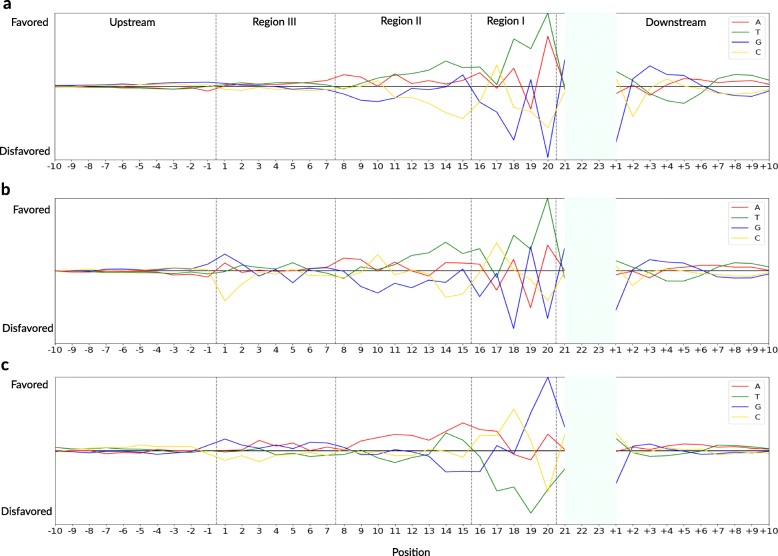
Base performance at 41 positions. The figure shows base preference of high on-target activity at 41 positions in **a** prokaryotic Cas9, **b** prokaryotic eSpCas9, and **c** eukaryotic scenarios

To understand invalidity across domains, we interpreted the trained CNN models and analyzed the learned biological features with perturbation-based approaches [46–48]. Figure 2 represents the base preference of high on-target activity at 41 positions in prokaryotic Cas9, prokaryotic eSpCas9, and a eukaryotic scenario.

Region I of the three scenarios shows extreme base preference and Region II takes second place (Fig. 2). We found enhanced base preference at position 20 in the three scenarios. In Region I, prokaryotic Cas9 and prokaryotic eSpCas9 favored T and A for high on-target activity, but positions 17 and 19 were two exceptions. In the eukaryotic scenario, preference in Region I showed a totally different pattern, where high on-target activity disfavored T and other base preferences represented a more complex landscape. In Region II, prokaryotic Cas9 and prokaryotic eSpCas9 base preferences changed smoothly, but shook at positions 10 and 15. In the eukaryotic scenario, high on-target activity favored A in Region II, while the T and G preferences changed at positions 14 and 15. In Region III, the eSpCas9 base preferences are more informative than Cas9, especially at position 1, where high on-target activity favored G and A and disfavored C. In upstream sites, high on-target activity favored G and C in the two prokaryotic scenarios, but they favored C and T in the eukaryotic scenario. In downstream sites, the preferences were reversed twice at positions +2 and +6 in all three scenarios. In addition, G was favored at position 21 and disfavored at position +1 in all three scenarios. Overall, two prokaryotic scenarios have the similar base preferences at 41 positions and they are different from the eukaryotic scenario.

**Fig. 3:**
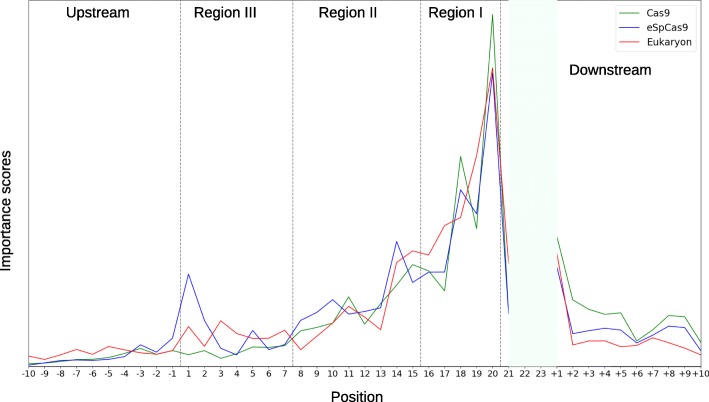
Importance scores at 41 positions. The figure shows the importance scores at 41 positions in prokaryotic Cas9, prokaryotic eSpCas9, and eukaryotic scenario. The normalized cumulative value of 41 points on each curve is one

We calculated importance scores by calculating the absolute values of four differences at each position. The normalized cumulative value of 41 points on each curve is shown in Fig. 3. The importance in downstream sequences was higher than upstream sequences. Torsional DNA constraints in flanking regions affect local target DNA strand shape, appropriate topological spatial conformation, and Cas9 cleavage complex binding to target DNA sites [41, 49]. Kim and co-workers suggested that 50 nt target sequence inputs performed better than 24, 27, and 34 nt-long inputs [42].

The predictive performance in prokaryotic eSpCas9 scenario is more accurate than prokaryotic Cas9 and eukaryotic scenarios. According to the ideology of transfer learning [50] and to improve performance, we fine-tuned the whole prokaryotic Cas9 model and eukaryotic model, which were both initialized with prokaryotic eSpCas9 model parameters. First, we rigorously removed overlapping samples. We found that the predictive performance for the prokaryotic Cas9 model was improved, but that the predictive performance of the eukaryotic model was not. Additional file 6: Figure S2 shows the prokaryotic Cas9 real-time average Spearman correlation coefficient changes under the same 5-fold cross-validation in the raw and transfer learning scenarios. The average Spearman correlation coefficients were improved from 0.5817 to 0.6279. Notably, the predictive correlation coefficient was 0.5822 using the eSpCas9 model within domains, suggesting fine-tuning is necessary.

### Potential off-target effects on on-target activity

**Fig. 4:**
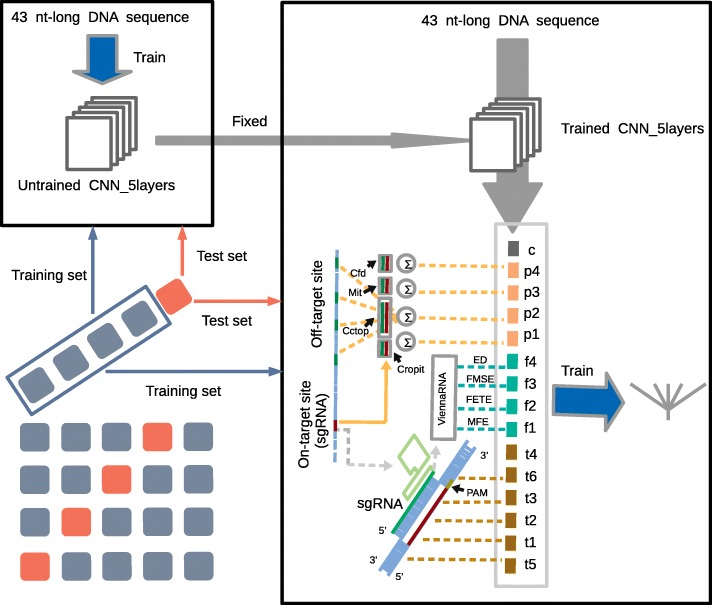
Flow chart of training CNN_5layers and further improving predictive performance under 5-fold cross-validation. The datasets were randomly and equally separated into five subgroups, and alternately four subgroups were used as the training set to train the models. The remaining subgroup was used to test the generalization capacity of the trained models. We combined trained CNN_5layers output with extra features to continue training simple linear regression models. Finally, the remaining subgroup was again used to test improved performances

To study potential off-target effects on sgRNA on-target activity, we calculated potential off-target scores (POS) for every sgRNA. Cropit_POS, Cctop_POS, Mit_POS, and Cfd_POS were potential off-target scores calculated by CROP-IT [51], CCTop [52], MITScore [20, 53], and CFDScore [22], respectively (see Methods and Fig. 4). It was reported that T(1_7), T(8_15), T(16_20) and T(1_20) were the top 4 scores [23] in all manually-extracted features, including position-independent features, position-specific features, PAM, GC content, and dinucleotide features. In comparison with melting temperatures, potential off-target scores are also important features for Cas9, eSpCas9, and Cas9 (△*recA*). We found that all Spearman correlation coefficients between the four POSs and sgRNA activity for eSpCas9 were lower than for Cas9 (Table 4), which could result from improved eSpCas9 specificity [54]. Comparing the four off-target predictors, MITScore [20, 53] seemed to be the most predictive. We used only four POSs as features (combination p in Table 5) to predict on-target activity by simple linear regression. The average Spearman correlation coefficient between predictive value and on-target activity was 0.1207 (0.1121) for Cas9 (eSpCas9) in Table 6. The Spearman correlation coefficient of combination p is also higher than p1, p2, p3, and p4, indicating that off-target potentiality affects on-target activity in the CRISPR-Cas9 system. Indeed, off-target activity and on-target activity interact with each other, and seems to be interpreted by molecular dynamics. If these off-target predictors can be further optimized, a higher degree of correlation is likely.

### Further performance improvement with additional features

Potential off-target scores involved genome-wide off-target alignments and evaluations, which could not be extracted from sgRNA and local target DNA sequences. Given that potential off-target scores are important extra features, we integrated CNN_5layers output and POSs to further improve predictive performance. Besides the POSs, we considered six melting temperatures and four RNA fold scores, including T(1_7), T(8_15), T(16_20), T(1_20), T(-5_-1), T(21_+2), MFE, FETE, FMSE, and ED. We tested several feature combinations. The feature combinations are described in Table 5. For CNN_5layers, the average test Spearman correlation coefficients are shown in Tables 2 and 6 (combination c). The training set correlations are shown in Table 6 (combination c). We used simple linear regression, regularized linear regression (L1LR and L2LR), support vector regression (SVR), and gradient boosting regression tree (GBR) with various parameters selection as predictor models. Under each cross-validation, we input previously-trained CNN_5layers output as new features (Fig. 4). We observed improved CNN_5layers predictions using the simple linear regression method (Table 6). Through internal comparisons (Table 4) and cross-comparisons between Table 4 and the top half of Table 6, we found that reciprocally combining POSs features, melting temperatures, and RNA fold scores achieved higher correlation coefficients. Regarding the bottom half of Table 6, adding POSs features, melting temperatures, or RNA fold scores to the CNN_5layers output (t_c, p_c, f_c) improved predictive performance in the training and test sets for Cas9 and eSpCas9. However, for Cas9 (△*recA*), only RNA fold scores (f_c) improved performance. In all three scenarios, melting temperatures did not greatly improve performance, indicating that CNN_5layers is more able to learn melting temperature features than RNA fold score features. Additionally, melting temperature features were extracted more easily (Additional file 1: Table S4). Collectively, we observed that the combination of POSs, RNA fold scores, and CNN_5layers output (p_f_c) achieved better predictive performance.

## Discussion

We compared our CNN_5layers with other published networks, such as CNN_Lin [44], DeepCas9 [42] and DeepCRISPR [29]. These models show different performance, indicating the need to analyze the cause of the differences. First, the number of parameters of CNN_Lin [44], DeepCas9 [42], and DeepCRISPR [29] are ∼22,000, ∼232,000, and ∼3,025,000 respectively. Our CNN_5layers has ∼190,000 parameters. Second, comparing DeepCas9 [42] with CNN_5layers, we found the number of parameters is roughly similar. However, DeepCas9 has three fully-connected layers with ∼223,000 parameters (proportion ∼96%). The proportion in CNN_5layers is only ∼7%, and batch normalization is used in our fully-connected layers. And another point is that our CNN_5layers addresses a larger sample space (43 nt-long input) than DeepCas9 (30 nt-long input). For DeepCas9, such large-scale, three fully-connected layers without batch normalization can fit many functions, but a few losses can propagate back to the convolution layers. The main advantage of CNN is abstracting features by convolution. Lin et al. [44] seemed to understand this situation, so they used a maximum pooling layer with a window size of 5 ×1 and stride 5 to downsize the fully-connected layer in their CNN_Lin. However, this down-sampling is not suitable for 23 ×1 size feature maps. Chuai et al. [29] used a fully convolutional network (FCN) without fully-connected layers. DeepCRISPR [29] has hundreds of channels, which can lead to severe over-fitting (Additional file 2: Figure S1). Third, target site flanking regions have heavily favored and unfavored nucleotides, especially in the downstream region. The DeepCas9 30 nt target DNA input size [42] (*N*_4_*N*_20_*NGGN*_3_) is so small that important feature information is omitted. As shown in Fig. 2, the region from + 4 to + 10 contains abundant preference information. Therefore, well-behaved network architectures need to be carefully and elaborately designed. We built several CNN architectures with multiple convolution layers and different scales of fully-connected layers (Additional file 3, Additional file 1: Table S1). CNN_2layers and CNN_3layers with large-scale fully-connected layers have relatively weak generalization ability, indicating that the number of convolution layers, channel size, and fully-connected layer scale should be adjusted and balanced carefully. Finally, we used our CNN_5layers architecture to predict sgRNA activity, surpassing available state-of-the-art models in both prokaryotes and eukaryotes.

Genome-wide potential off-target effects influence on-target activity and utilized genome-wide accumulative potential off-target scores to further improve predictive performance for Cas9, eSpCas9 and Cas9 (△*recA*). However, the performance improvements are very small. Multi-modal network architectures have been widely reported, so we attempted to make POSs a CNN input by adding them as an input channel or concatenating them into flattened fully-connected layers. However, fluctuations in predictive performance made it difficult to determine exactly whether performance improved. Our two-step training method shows improved performance. The main advantage of CNN is abstracting features layer by layer [55]. We adopted batch normalization and down-sampling to avoid over-fitting in the training set, which allowed the training set to be reused to train other models with additional features.

Next, we used perturbation-based approaches to analyze single-base preference at certain positions. We also analyzed dinucleotide, trinucleotide, and more complex biological pattern preference. We believe that our CNN could learn RNA folding features. However, it seems difficult to interpret long-range interactions that CNN learns. In the future, we will consider a more powerful model [47, 48, 56, 57] to improve long-range interaction prediction. It is unclear which biological factors cause different patterns between prokaryotic and eukaryotic editing. Many factors have effects on gene editing activity, such as guide sequence folding, off-target effects, chromatin structure, simple cutting activity, and double strand break (DSB) repair. For example, dense chromatin structures could introduce noise in data from eukaryotic cells [30, 32, 33]. Bacteria mainly rely on homologous recombination (HR) with sister chromosomes to repair DSBs [40], instead of nonhomologous end joining (NHEJ). Our prokaryotic on-target activity is calculated by cell toxicity induced by DSB. Eukaryotic on-target activity is based on toxicity or base indels. Different DSB end configurations are important for eukaryotic NHEJ efficiency [58]. Guo et al. [23] posits that NHEJ can also introduce noise in eukaryotic cell data. In most cases, the cutting site is between positions 17 and 18. We found position preference from 15 to 20 nt was different between prokaryotic and eukaryotic scenarios than other positions (Fig. 2). These differences may result from joining efficiency and NHEJ preference. Several other biological factors contribute to the different patterns between prokaryotic and eukaryotic editing, such as conformation changes and internal solvation kinetics of the Cas9 protein. The cause of these differences will be investigated in future studies.

CROP-IT [51], CCTop [52], MITScore [20, 53], and CFDScore [22] were used to calculate POSs for every sgRNA. These packages can still be improved. In living cells, off-target and on-target activity influence each other. Thus, it is possible that on-target and off-target prediction models will be optimized together.

## Conclusion

We conclude that deep neural networks can improve the predictive performance of sgRNA on-target activity in prokaryotes. By increasing the number of network convolution layers and target DNA sequence input size, we developed a CNN with five convolution layers to predict sgRNA activity. Our network outperformed state-of-the-art traditional machine learning algorithms and other CNN models. We confirmed that sgRNA activity prediction models trained on prokaryotes not appropriate for eukaryotes. We trained our CNN_5layers network based eukaryotic data, similarly surpassing available state-of-the-art eukaryotic models. Thus, our CNN_5layers network has certain generalization ability and has improved performance in eukaryotes. We used perturbation-based approaches to analyze different biological patterns between prokaryotic and eukaryotic editing. Then, we improved the predictive performance of prokaryotic Cas9 by transfer learning. Finally, we confirmed that genome-wide potential off-target effects and sgRNA guide sequence folding have effects on on-target activity. We also used genome-wide accumulative potential off-target scores and RNA fold scores to further improve predictive performance. We believe that our algorithm can also be applied to eukaryotes.

## Methods

### Benchmark datasets

The main datasets that we used in training and testing were from ∼70,000 sgRNA activity maps, which were systematically profiled by co-expressing a genome-scale library with a pooled screening strategy in *Escherichia coli*. The high-quality datasets were established for Cas9 (*Streptococcus pyogenes*), eSpCas9, and Cas9 (△*recA*). The eSpCas9 is a reengineered Cas9 derivative with improved specificity, containing K810A, K1003A, and R1060A mutations [54]. Cas9 (△*recA*) was developed in *Escherichia coli* by knockout of *recA* blocking DSBs repair. Three datasets included 44,163, 45,070, and 48,112 sgRNAs, respectively [23].

### Extending sequences

We mapped these 20 nt sgRNA guide sequences to the *Escherichia coli* reference genome (K12 MG1655, NC000913.3), ensuring that target regions were flanked by a 3’NGG PAM site. Then, we extended the target DNA sequences to 43 nt, namely *N*_10_*N*_20_*NGGN*_10_ (*N* represents any nucleotide, and the first and last 10 nt are the extended portions). There were several reasons for these extensions: (1) some studies indicate that sequences upstream and downstream of sgRNA target sites have favored and unfavored nucleotides in human and mouse cells [21, 24], especially enrichment or depletion in flanking regions in *Ciona* cells [59]; (2) the deep learning algorithm has unique advantages in automatic feature extraction with noise interference [60–62]; (3) it is possible that occupation by nucleoid-associated proteins, transcription factor binding, and torsional constraints in flanking regions sterically hinder Cas9 cleavage complex binding and local DNA strand movement to appropriate conformations [41, 49]. Deep learning algorithms will detect these hidden factors. We found three 20 nt guide sequences mapped multiple times to reference genome. Nevertheless, we retained these ambiguous mapping samples to prevent changing sample distribution for comparisons with previous algorithms. After sequence extension, we established three datasets (Set 1; see Table 1 and Additional file 7).

### Removing redundancy

There were no overlapping samples within any dataset. However, we routinely reduced sequence redundancy using CD-HIT [63, 64]. Removing highly similar sequences can reduce natural sample-biased effects and make models more robust, especially when similar samples occur between training and test datasets, causing an illusion of good performance. We found that some sgRNAs target adjacent DNA sites in Set 1. For pooled screening, these adjacent samples could have mutual interference, resulting in low-quality data. Therefore, we used CD-HIT to remove redundancy in Set 1 with similarity threshold 0.8, which established dataset Set 2, also including Cas9, eSpCas9 and Cas9 (△*recA*) (Table 1).

### Model establishing

For model inputs, we converted 43 nt-long DNA sequences into one hot code representation. Each position had a four-element vector with one component set to one and the others set to zero. Each sample was represented as a 4 ×43×1 three-dimensional matrix, where 4 represents four channels. The model output is a value which predicts on-target activity.

To achieve the desired performance, we adopted advanced algorithms such as batch normalization and a leaky rectified linear unit (LeakyReLU). Batch normalization (BN) addresses the internal covariate shift. This method normalizes each training mini-batch, allows much higher learning rates, and makes model more easily initiated [65]. The ReLU was proposed for deep learning in 2015 [66], since it is more biologically plausible, and can act as the activation function for hidden units. Nevertheless, the ReLU cannot learn via gradient-based methods when activation values are zero. In this study, we used a LeakyReLU, which allows a small gradient when the unit is not activated. Some studies demonstrated that LeakyReLU is a better activation function, and may replace traditional activation functions [67, 68]. Therefore, we constructed convolution layers with a convolution operation, batch normalization, and a leaky rectified linear unit (Fig. 1a).

In this study, we found that factors that affecting model performance include the number of convolution layers, channel size, and scale of fully-connected layers. Therefore, we utilized convolution layers with a *n*×3×1 and stride 1 convolution kernel size, a maximum pooling layer with 2 ×1 and stride 2 window size, and two fully-connected layers to build CNNs with various convolution layers and multiple scales of fully-connected layers (Additional file 3). Finally, we trained and tested these networks for model selection and discussed the merits and disadvantages of network architectures in the discussion section.

### Other models for comparison

We compared our algorithms with three recently published prediction algorithms based on deep learning. The first network architecture is DeepCRISPR [29], which is a fully convolutional neural network (FCN) and predicts on-target knockout efficacy in eukaryotic cells with better performance than the available state-of-the-art tools. The second network architecture was meticulously designed as an off-target predictive classifier in eukaryotic cells [44]. Because the off-target input size matched perfectly with on-target input size (23 nt), we retrained it using our on-target dataset. The difference is that we changed the output size of the last fully-connected layer to one value instead of two values and changed the binary cross entropy loss function to the mean squared error. We named this modified network architecture CNN_Lin. Similar to CNN_Lin, DeepCas9 [42] is a convolutional neural network with only one convolution layer. The difference is that the DeepCas9 input size is 30 nt-long target DNA sequences (*N*_4_*N*_20_*NGGN*_3_) [42]. Another convolutional neural network based on only convolution layer and 30 nt-long inputs is also named DeepCas9 [43]. We distinguished between DeepCas9 [42] and DeepCas9 [43] by reference annotation. Thus, we can compare the performances of 30 nt-long input with 23 nt-long input. It is worth noting that DeepCRISPR [29], CNN_Lin [44], DeepCas9 [42], and our CNNs were all trained and tested with the same training dataset, test dataset, and separation of 5-fold cross-validation. We adopted mean squared error as the loss function of these CNN regression models. The last layer of all CNN architectures does not contain a batch normalization and activation function to avoid limiting the numerical output range.

### Independent eukaryotic datasets

We used fourteen eukaryotic datasets which were collected and arranged by Haeussler et al. [53]. We chose three high-quality datasets (xu2015TrainHl60_Kbm7, doench2014Hs, doench2014Mm) from the fourteen eukaryotic datasets to train a eukaryotic model. The remaining eleven datasets (Table 3) were used to independently test eukaryotic models. We normalized the on-target activity values of each eukaryotic dataset and concatenated the three datasets as a eukaryotic training set. Therefore, the eukaryotic on-target values vary between zero and one, which is different from the three prokaryotic model on-target value ranges (Table 1). This was also necessary to compare our eukaryotic model with other models. We compared our model with DeepCRISPR [29], DeepCas9 [43], and TSAM [28]. Unlike comparisons in the prokaryotic models, we did not need to retrain the networks with similar data, because of the eleven independent eukaryotic test sets. To ensure valid comparisons, we had to remove some overlapping samples in each model training set for the eleven eukaryotic datasets. Finally, to verify the validity of prokaryotic models for eukaryotes, we used the remaining eleven datasets to test prokaryotic trained models. The eukaryotic training set and eleven independent eukaryotic test sets are described in Additional file 4.

### Melting temperatures and RNA fold scores

Previous studies showed that the predictive scores were most influenced by melting temperature, which is determined by Watson-Crick base pairing. In other words, the melting temperatures in different regions are effective predictive metrics [23, 28]. We computed the melting temperatures from different target site regions using the Biopython Tm_stalus function [69, 70]. We needed to number *N*_10_*N*_20_*NGGN*_10_ in a certain order, -10 to -1 for the first *N*_10_ (upstream), 1 to 23 for *N*_20_*NGG*, and +1 to +10 for the last *N*_10_ (downstream). Then, we calculated the melting temperatures of 1 to 7 (Region III), 8 to 15 (Region II), 16 to 20 (Region I), 1 to 20, -5 to -1, and 21 to +2 (T(1_7), T(8_15), T(16_20), T(1_20), T(-5_-1), and T(21_+2), respectively; Table 4). Guide sequences (20 nt) within the 5’ end of crRNA-tracrRNA duplexes could form secondary structures [1, 4], which could have adverse effects on sgRNA activity. We used ViennaRNA [45] to predict secondary structures of 20 nt-long RNA fragments (Fig. 4). We used four physicochemical properties to characterize RNA folding, including minimum free energy (MFE), free energy of the thermodynamic ensemble (FETE), frequency of the minimum free energy structure in the ensemble (FMSE), and ensemble diversity (ED) (Additional file 8).

### Potential off-target scores

Off-target effects were detected in multiple cell types. Off-target effects possibly happen at any region on a genome-wide scale as long as the region contains a PAM and 20 nt-long protospacer sequence with minor mismatch. Different sgRNAs have different number of candidate off-target sites. Moreover, different candidate off-target sites have different off-target efficiencies for a given sgRNA. To research potential off-target influence on on-target activity for various sgRNAs, we found out genome-wide potential off-target sites (allowing up to six mismatches) for every sgRNA in *Escherichia coli* by Cas-OFFinder [71]. We found that, on average, each sgRNA has 24 candidate off-target sites. We used CROP-IT [51], CCTop [52], MITScore [20, 53], and CFDScore [22] to respectively calculate off-target score of each candidate off-target site for certain sgRNA (Fig. 4). The four off-target predictors independently devised heuristics based on the distances of the mismatches to the PAM [53] and did not contain too many parameters. Finally, we respectively accumulated potential off-target scores (POS) of all candidate off-target sites for every sgRNA (Additional file 9).

### Training methodology and parameters

We trained our CNNs and other networks on a single NVIDIA Quadra P6000 GPU. Our CNNs were based on PyTorch framework. For DeepCRISPR [29], DeepCas9 [42] and CNN_Lin [44], we adopted published TensorFlow source codes. DeepCas9 [43] was based on MXNet, which had been gradually abandoned, so we only used DeepCas9 [42]. All networks were trained using the Adam optimizer with initial learning rate of 0.001, and default hyper-parameters *β*_1_ = 0.9, *β*_2_ = 0.999, *ε* = 1e - 08. The batch size is set to 128. As shown in Fig. 4, we randomly and equally separated the dataset into five subgroups, and alternately four subgroups were used as the training set to train the models. The remaining subgroup was used to test the generalization capacity of the trained models. Then, we combined trained CNN_5layers output with extra features to continue training a traditional machine learning model to improve prediction. Finally, the remaining subgroup was again used to test performances (Fig. 4).

### Interpretability and transfer learning

We adopted perturbation-based approaches, which changed a part of the input and observed its impact on the model output [46–48]. We used trained model to predict all samples and accumulate their predictive values (PSA). Then, we converted the nucleotide to A, T, C, and G at certain position. For example, the nucleotide at position -10 can be converted to A, T, C, and G respectively for all samples. Similarly, we used trained model to predict all samples-changed and accumulate their predictive values (PSAc). We had to carry out 41 ×4 PSAc with the fixed GG in NGG. The difference between PSAc and PSA can indicate that the substitute is favored (positive difference) or disfavored (negative difference) to high on-target activity. We also calculated the importance score at each position by accumulating the absolute values of its four differences. In addition, we fine-tuned the whole prokaryotic Cas9 and eukaryotic model, which were both initialized with the prokaryotic eSpCas9 model parameter, expecting to improve performance. It is necessary to remove the samples from test set, which has the same 43 nt-long feature sequences with eSpCas9 training samples. Otherwise, the improved performance is unconvincing. It is also important to convert the on-target activity value range of two training sets to output range of prokaryotic eSpCas9 model. We adopted previous training methodology including Adam optimizer and learning rate, loss function and separating of 5-fold cross-validation (Fig. 4). The prokaryotic Cas9 (△*recA*) was not involved in research of models interpretability and transfer learning.

### Performance evaluation and statistical significance

We used the Spearman correlation coefficient to evaluate the model performance, which is defined as

$$r = 1 - \frac{6\sum_{i}d_{i}^{2}}{n(n^{2}-1)} $$

where *n* is the number of data points of the two variables and *d*_*i*_ is the difference in the ranks of the *i*^*th*^ element of each random variable considered. The two variables are on-target activity value and its prediction. We used *t*-test to test the Spearman correlation coefficients. We calculated the Spearman correlation coefficients and the *p*-values by SciPy library in Python. Moreover, we used a Steiger test to compare the Spearman coefficients between two models, which was performed by psych package in R.

## Supplementary information

**Additional file 1** More detailed tables. Statistical significance of Spearman correlation coefficients in this paper and more details about 5-fold cross-validation. **Table S1.** Performance of all modes including DeepCRISPR, CNN_Lin, DeepCas9, CNN_2layers, CNN_3layers, CNN_4layers, CNN_5layers, and CNN_7layers. **Table S2.** The Spearman correlation coefficients and significances between eukaryotic sgRNA activity and predictions based on our prokaryotic models and eukaryotic models. **Table S3.** Significance between on-target activity and six melting temperatures, four RNA fold scores, and four POSs. **Table S4.** Detailed information of 5-fold cross-validation for several network architectures.

**Additional file 2** **Figure S1.** Real-time performance comparison under 5-fold cross-validation. The figure shows the real-time average Spearman correlation coefficients changes during being trained for several network architectures. Horizontal *x*-axis is training epochs, and vertical *y*-axis is average test Spearman correlation coefficients under 5-fold cross-validation. **a**, **b** and **c** represent Cas9, eSpCas9 and Cas9 (△*recA*), respectively. The light pink dashed are respectively corresponding to Spearman correlation coefficients of 0.542, 0.682 and 0.328 for Cas9, eSpCas9 and Cas9 (△*recA*), which are performances of gradient boosting regression trees.

**Additional file 3** CNN architectures for comparison. CNN architectures from CNN_2layers to CNN_7layers.

**Additional file 4** Eukaryotic datasets. Eleven independent eukaryotic test and training set of DeepCRISPR, DeepCas9, TSAM, and our CNN_5layers.

**Additional file 5** Melting temperatures. The melting temperatures of 43 nt-long target DNA sequences.

**Additional file 6** **Figure S2.** Real-time performance comparison between raw and transfer learning scenario. The figure shows the real-time average Spearman correlation coefficients changes during being trained in raw and transfer learning scenario. Horizontal *x*-axis is training epochs, and vertical *y*-axis is average test Spearman correlation coefficients under 5-fold cross-validation.

**Additional file 7** Set 1 dataset. Set 1 samples including 44,163, 45,070, and 48,112 sgRNAs for Cas9, eSpCas9 and Cas9 (△*recA*), respectively.

**Additional file 8** RNA fold scores. Four physicochemical properties characterizing 20 nt-long guide sequences fold.

**Additional file 9** All sgRNAs POSs. POSs calculated by CROP-IT, CCTop, MITScore, and CFD-Score.

## Data Availability

Source code is freely available at https://github.com/biomedBit/DeepSgrnaBacteria. The source code repository includes software application, detailed user manual and all relevant data.

## Abbreviations

BN: Batch Normalization
CNN: Convolution Neural Network
DSB: Double-stranded Break
ED: Ensemble Diversity
FETE: Free Energy of the Thermodynamic Ensemble
FMSE: Frequency of the Minimum free energy Structure in the Ensemble
GBR: Gradient Boosting Regression tree
HR: Homologous Recombination
LeakyReLU: Leaky Rectified Linear Unit
MFE: Minimum Free Energy
NHEJ: Nonhomologous End Joining
PAM: Protospacer Adjacent Motif
POS: Potential Off-target Score
PSA: Predict all Samples and Accumulate their predictive values
PSAc: Predict all Samples-changed and Accumulate their predictive values ReLU: Rectified Linear Unit
sgRNA: Single-guide RNA
tracrRNA: Trans-activating RNA

## References

[1] Deltcheva E, Chylinski K, Sharma CM, Gonzales K, Chao YJ, Pirzada ZA, Eckert MR, Vogel J, Charpentier E (2011). Crispr rna maturation by trans-encoded small rna and host factor rnase iii. Nature.

[2] Mali P, Yang L, Esvelt KM, Aach J, Guell M, DiCarlo JE, Norville JE, Church GM (2013). Rna-guided human genome engineering via cas9. Science.

[3] Cong L, Ran FA, Cox D, Lin SL, Barretto R, Habib N, Hsu PD, Wu XB, Jiang WY, Marraffini LA, Zhang F (2013). Multiplex genome engineering using crispr/cas systems. Science.

[4] Mojica FJM, Diez-Villasenor C, Garcia-Martinez J, Almendros C (2009). Short motif sequences determine the targets of the prokaryotic crispr defence system. Microbiol-Sgm.

[5] Sternberg SH, Redding S, Jinek M, Greene EC, Doudna JA (2014). Dna interrogation by the crispr rna-guided endonuclease cas9. Nature.

[6] Anders C, Niewoehner O, Duerst A, Jinek M (2014). Structural basis of pam-dependent target dna recognition by the cas9 endonuclease. Nature.

[7] Jinek M, Chylinski K, Fonfara I, Hauer M, Doudna JA, Charpentier E (2012). A programmable dual-rna-guided dna endonuclease in adaptive bacterial immunity. Science.

[8] Bolukbasi MF, Gupta A, Wolfe SA (2016). Creating and evaluating accurate crispr-cas9 scalpels for genomic surgery. Nat Methods.

[9] Shalem O, Sanjana NE, Hartenian E, Shi X, Scott DA, Mikkelsen TS, Heckl D, Ebert BL, Root DE, Doench JG, Zhang F (2014). Genome-scale crispr-cas9 knockout screening in human cells. Science.

[10] Swiech L, Heidenreich M, Banerjee A, Habib N, Li Y. Q, Trombetta J, Sur M, Zhang F (2015). In vivo interrogation of gene function in the mammalian brain using crispr-cas9. Nat Biotechnol.

[11] Hart T, Brown KR, Sircoulomb F, Rottapel R, Moffat J (2014). Measuring error rates in genomic perturbation screens: gold standards for human functional genomics. Mol Syst Biol.

[12] Konermann S, Brigham MD, Trevino AE, Joung J, Abudayyeh OO, Barcena C, Hsu PD, Habib N, Gootenberg JS, Nishimasu H, Nureki O, Zhang F (2015). Genome-scale transcriptional activation by an engineered crispr-cas9 complex. Nature.

[13] Gilbert LA, Horlbeck MA, Adamson B, Villalta JE, Chen Y, Whitehead EH, Guimaraes C, Panning B, Ploegh HL, Bassik MC, Qi LS, Kampmann M, Weissman JS (2014). Genome-scale crispr-mediated control of gene repression and activation. Cell.

[14] Shapiro RS, Chavez A, Porter CBM, Hamblin M, Kaas CS, DiCarlo JE, Zeng G, Xu X, Revtovich AV, Kirienko NV, Wang Y, Church GM, Collins JJ (2018). A crispr-cas9-based gene drive platform for genetic interaction analysis in candida albicans. Nat Microbiol.

[15] Shen JP, Zhao D, Sasik R, Luebeck J, Birmingham A, Bojorquez-Gomez A, Licon K, Klepper K, Pekin D, Beckett AN, Sanchez KS, Thomas A, Kuo CC, Du D, Roguev A, Lewis NE, Chang AN, Kreisberg JF, Krogan N, Qi L, Ideker T, Mali P (2017). Combinatorial crispr-cas9 screens for de novo mapping of genetic interactions. Nat Methods.

[16] Hart T, Chandrashekhar M, Aregger M, Steinhart Z, Brown KR, MacLeod G, Mis M, Zimmermann M, Fradet-Turcotte A, Sun S, Mero P, Dirks P, Sidhu S, Roth FP, Rissland OS, Durocher D, Angers S, Moffat J (2015). High-resolution crispr screens reveal fitness genes and genotype-specific cancer liabilities. Cell.

[17] Smith C, Abalde-Atristain L, He C, Brodsky BR, Braunstein EM, Chaudhari P, Jang YY, Cheng L, Ye Z (2015). Efficient and allele-specific genome editing of disease loci in human ipscs. Mol Ther.

[18] Cox DBT, Platt RJ, Zhang F (2015). Therapeutic genome editing: prospects and challenges. Nat Med.

[19] Yin C, Zhang T, Qu X, Zhang Y, Putatunda R, Xiao X, Li F, Xiao W, Zhao H, Dai S, Qin X, Mo X, Young WB, Khalili K, Hu W (2017). In vivo excision of hiv-1 provirus by sacas9 and multiplex single-guide rnas in animal models. Mol Ther.

[20] Hsu PD, Scott DA, Weinstein JA, Ran FA, Konermann S, Agarwala V, Li Y, Fine EJ, Wu X, Shalem O, Cradick TJ, Marraffini LA, Bao G, Zhang F (2013). Dna targeting specificity of rna-guided cas9 nucleases. Nat Biotechnol.

[21] Doench JG, Hartenian E, Graham DB, Tothova Z, Hegde M, Smith I, Sullender M, Ebert BL, Xavier RJ, Root DE (2014). Rational design of highly active sgrnas for crispr-cas9-mediated gene inactivation. Nat Biotechnol.

[22] Doench JG, Fusi N, Sullender M, Hegde M, Vaimberg EW, Donovan KF, Smith I, Tothova Z, Wilen C, Orchard R (2016). Optimized sgrna design to maximize activity and minimize off-target effects of crispr-cas9. Nat Biotechnol.

[23] Guo J, Wang T, Guan C, Liu B, Luo C, Xie Z, Zhang C, Xing XH. Improved sgrna design in bacteria via genome-wide activity profiling. Nucleic Acids Res. 2018; 46(14):7052–69. 10.1093/nar/gky572. 10.1093/nar/gky572 PMC6101607 29982721

[24] Xu H, Xiao T, Chen CH, Li W, Meyer CA, Wu Q, Wu D, Cong L, Zhang F, Liu JS, Brown M, Liu XS (2015). Sequence determinants of improved crispr sgrna design. Genome Res.

[25] Chari R, Mali P, Moosburner M, Church G. M (2015). Unraveling crispr-cas9 genome engineering parameters via a library-on-library approach. Nat Methods.

[26] Chari R, Yeo NC, Chavez A, Church GM (2017). sgrna scorer 2.0: A species-independent model to predict crispr/cas9 activity. ACS Synth Biol.

[27] Moreno-Mateos MA, Vejnar CE, Beaudoin JD, Fernandez JP, Mis EK, Khokha MK, Giraldez AJ (2015). Crisprscan: designing highly efficient sgrnas for crispr-cas9 targeting in vivo. Nat Methods.

[28] Peng H, Zheng Y, Blumenstein M, Tao D, Li J (2018). Crispr/cas9 cleavage efficiency regression through boosting algorithms and markov sequence profiling. Bioinformatics.

[29] Chuai G, Ma H, Yan J, Ming C, Hong N, Xue D, Chi Z, Zhu C, Ke C, Duan B. Deepcrispr : optimized crispr guide rna design by deep learning. Genome Biol. 2018; 19(1):80. 10.1186/s13059-018-1459-4. 10.1186/s13059-018-1459-4 PMC6020378 29945655

[30] Uusi-Mäkelä MIE, Barker HR, Bäuerlein CA, Häkkinen T, Nykter M, Rämet M. Chromatin accessibility is associated with CRISPR-Cas9 efficiency in the zebrafish (Danio rerio). PloS ONE. 2018; 13(4):e0196238. 10.1371/journal.pone.0196238. 10.1371/journal.pone.0196238 PMC5912780 29684067

[31] Wu X, Scott DA, Kriz AJ, Chiu AC, Hsu PD, Dadon DB, Cheng AW, Trevino AE, Konermann S, Chen S, Jaenisch R, Zhang F, Sharp PA (2014). Genome-wide binding of the crispr endonuclease cas9 in mammalian cells. Nat Biotechnol.

[32] Yarrington RM, Verma S, Schwartz S, Trautman JK, Carroll D (2018). Nucleosomes inhibit target cleavage by crispr-cas9 in vivo. Proc Natl Acad Sci U S A.

[33] Horlbeck MA, Witkowsky LB, Guglielmi B, Replogle JM, Gilbert LA, Villalta JE, Torigoe SE, Tjian R, Weissman JS (2016). Nucleosomes impede cas9 access to dna in vivo and in vitro. Elife.

[34] Volkov A, Mascarenhas J, Andrei-Selmer C, Ulrich HD, Graumann PL (2003). A prokaryotic condensin/cohesin-like complex can actively compact chromosomes from a single position on the nucleoid and binds to dna as a ring-like structure. Mol Cell Biol.

[35] Mendoza-Vargas A, Olvera L, Olvera M, Grande R, Vega-Alvarado L, Taboada B, Jimenez-Jacinto V, Salgado H, Juárez K, Contreras-Moreira B, Huerta AM, Collado-Vides J, Morett E (2009). Genome-wide identification of transcription start sites, promoters and transcription factor binding sites in e. coli. PLoS ONE.

[36] Struhl K (1999). Fundamentally different logic of gene regulation in eukaryotes and prokaryotes. Cell.

[37] Garst AD, Bassalo MC, Pines G, Lynch SA, Halweg-Edwards AL, Liu RM, Liang LY, Wang ZW, Zeitoun R, Alexander WG, Gill RT (2017). Genome-wide mapping of mutations at single-nucleotide resolution for protein, metabolic and genome engineering. Nat Biotechnol.

[38] Tong YJ, Charusanti P, Zhang LX, Weber T, Lee SY (2015). Crispr-cas9 based engineering of actinomycetal genomes. ACS Synth Biol.

[39] Zerbini F, Zanella I, Fraccascia D, Konig E, Irene C, Frattini LF, Tomasi M, Fantappie L, Ganfini L, Caproni E, Parri M, Grandi A, Grandi G (2017). Large scale validation of an efficient crispr/cas-based multi gene editing protocol in escherichia coli. Microb Cell Fact.

[40] Cui L, Bikard D (2016). Consequences of cas9 cleavage in the chromosome of escherichia coli. Nucleic Acids Res.

[41] Farasat I, Salis HM (2016). A biophysical model of crispr/cas9 activity for rational design of genome editing and gene regulation. PLoS Comput Biol.

[42] Kim HK, Min S, Song M, Jung S, Choi JW, Kim Y, Lee S, Yoon S, Kim HH (2018). Deep learning improves prediction of crispr-cpf1 guide rna activity. Nat Biotechnol.

[43] Xue L, Tang B, Chen W, Luo JS (2019). Prediction of crispr sgrna activity using a deep convolutional neural network. J Chem Inf Model.

[44] Lin J, Wong K-C (2018). Off-target predictions in crispr-cas9 gene editing using deep learning. Bioinformatics.

[45] Lorenz R, Bernhart SH, Siederdissen CHZ, Tafer H, Flamm C, Stadler PF, Hofacker IL (2011). Viennarna package 2.0. Algoritm Mol Biol.

[46] Li Y, Huang C, Ding L, Li Z, Pan Y, Gao X. Deep learning in bioinformatics: Introduction, application, and perspective in the big data era. Methods. 2019; 166:4–21. 10.1016/j.ymeth.2019.04.008. 10.1016/j.ymeth.2019.04.008 31022451

[47] Umarov R, Kuwahara H, Li Y, Gao X, Solovyev V. Promoter analysis and prediction in the human genome using sequence-based deep learning models. Bioinformatics. 2019; 35(16):2730–7. 10.1093/bioinformatics/bty1068. 10.1093/bioinformatics/bty1068 30601980

[48] Dai HJ, Umarov R, Kuwahara H, Li Y, Song L, Gao X (2017). Sequence2vec: a novel embedding approach for modeling transcription factor binding affinity landscape. Bioinformatics.

[49] Räz MH, Hidaka K, Sturla SJ, Sugiyama H, Endo M (2016). Torsional constraints of dna substrates impact cas9 cleavage. J Am Chem Soc.

[50] Tang BH, Pan ZX, Yin K, Khateeb A (2019). Recent advances of deep learning in bioinformatics and computational biology. Front Genet.

[51] Singh R, Kuscu C, Quinlan A, Qi Y, Adli M (2015). Cas9-chromatin binding information enables more accurate crispr off-target prediction. Nucleic Acids Res.

[52] Stemmer M, Thumberger T, Del Sol Keyer M, Wittbrodt J, Mateo JL (2015). Cctop: An intuitive, flexible and reliable crispr/cas9 target prediction tool. PLoS ONE.

[53] Haeussler M, Kai S, Eckert H, Eschstruth A, Mianné J, Renaud JB, Schneider-Maunoury S, Shkumatava A, Teboul L, Kent J. Evaluation of off-target and on-target scoring algorithms and integration into the guide rna selection tool crispor. Genome Biol. 2016; 17(1):148. 10.1186/s13059-016-1012-2. 10.1186/s13059-016-1012-2 PMC4934014 27380939

[54] Slaymaker IM, Gao L, Zetsche B, Scott DA, Yan WX, Zhang F (2016). Rationally engineered cas9 nucleases with improved specificity. Science.

[55] Szegedy C, Liu W, Jia YQ, Sermanet P, Reed S, Anguelov D, Erhan D, Vanhoucke V, Rabinovich A. Going deeper with convolutions. In: 2015 IEEE Conference on Computer Vision and Pattern Recognition (CVPR). IEEE: 2015. 10.1109/cvpr.2015.7298594.

[56] Zhang J, Peng W, Wang L (2018). Lenup: learning nucleosome positioning from dna sequences with improved convolutional neural networks. Bioinformatics.

[57] Lyu C, Wang L, Zhang J (2018). Deep learning for dnase i hypersensitive sites identification. BMC Genomics.

[58] Chang HHY, Watanabe G, Gerodinnos CA, Ochi T, Blundell TL, Jackson SP, Lieber MR (2016). Different dna end configurations dictate which nhej components are most important for joining efficiency. J Biol Chem.

[59] Gandhi S, Haeussler M, Razy-Krajka F, Christiaen L, Stolfi A (2017). Evaluation and rational design of guide rnas for efficient crispr/cas9-mediated mutagenesis in ciona. Dev Biol.

[60] Koh PW, Pierson E, Kundaje A (2017). Denoising genome-wide histone chip-seq with convolutional neural networks. Bioinformatics.

[61] Ghifary M, Kleijn WB, Zhang MJ. Sparse representations in deep learning for noise-robust digit classification. In: 2013 28th International Conference on Image and Vision Computing New Zealand (IVCNZ 2013). IEEE: 2013. 10.1109/ivcnz.2013.6727040.

[62] Edwards C (2018). Deep learning hunts for signals among the noise. Commun ACM.

[63] Li W, Godzik A (2006). Cd-hit: a fast program for clustering and comparing large sets of protein or nucleotide sequences. Bioinformatics.

[64] Fu L, Niu B, Zhu Z, Wu S, Li W (2012). Cd-hit: accelerated for clustering the next-generation sequencing data. Bioinformatics.

[65] Ioffe S, Szegedy C. Batch normalization: Accelerating deep network training by reducing internal covariate shift. In: ICML’15 Proceedings of the 32nd International Conference on International Conference on Machine Learning - Volume 37. Lille: 2015. p. 448–456. https://dl.acm.org/citation.cfm?id=3045118.3045167.

[66] Hara K, Saito D, Shouno H. Analysis of function of rectified linear unit used in deep learning. In: 2015 International Joint Conference on Neural Networks (IJCNN). IEEE: 2015. 10.1109/ijcnn.2015.7280578.

[67] Habibi Aghdam H, Jahani Heravi E, Puig D. Toward an optimal convolutional neural network for traffic sign recognition. In: Eighth International Conference on Machine Vision (ICMV 2015). SPIE: 2015. 10.1117/12.2228582.

[68] Zhang Y, Hou X, Chen Y, Chen H, Yang M, Yang J, Wang S (2018). Voxelwise detection of cerebral microbleed in cadasil patients by leaky rectified linear unit and early stopping. Multimedia Tools Appl.

[69] Cock PJA, Antao T, Chang JT, Chapman BA, Cox CJ, Dalke A, Friedberg I, Hamelryck T, Kauff F, Wilczynski B, de Hoon MJL (2009). Biopython: freely available python tools for computational molecular biology and bioinformatics. Bioinformatics.

[70] Le Novere N (2001). Melting, computing the melting temperature of nucleic acid duplex. Bioinformatics.

[71] Bae S, Park J, Kim JS (2014). Cas-offinder: a fast and versatile algorithm that searches for potential off-target sites of cas9 rna-guided endonucleases. Bioinformatics.

