# Supplementary figures and images for: Prediction of sgRNA on-target activity in bacteria by deep learning
Source: BMC Bioinformatics. 2019 Oct 24;20:517. doi: 10.1186/s12859-019-3151-4 (PMC6814057; doi:10.1186/s12859-019-3151-4)

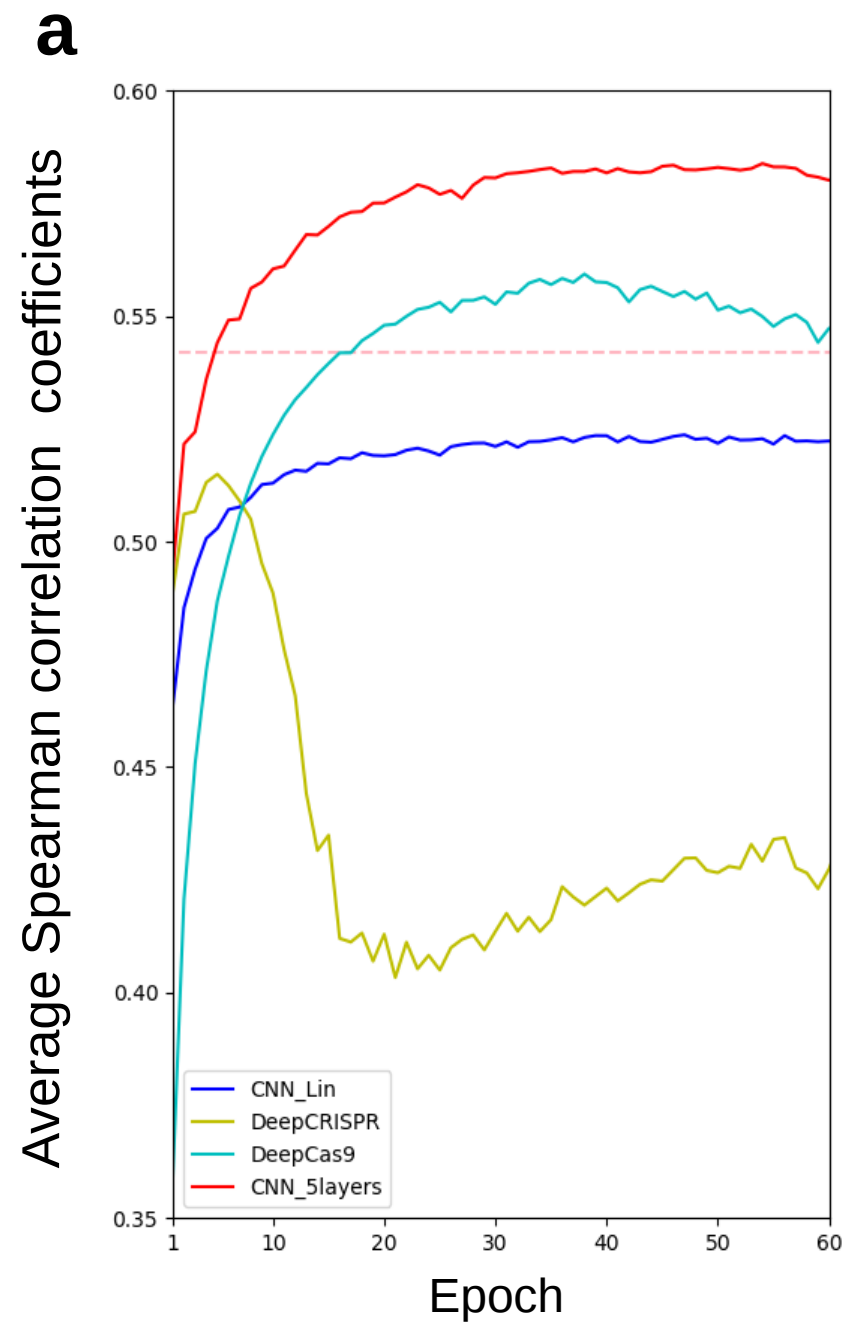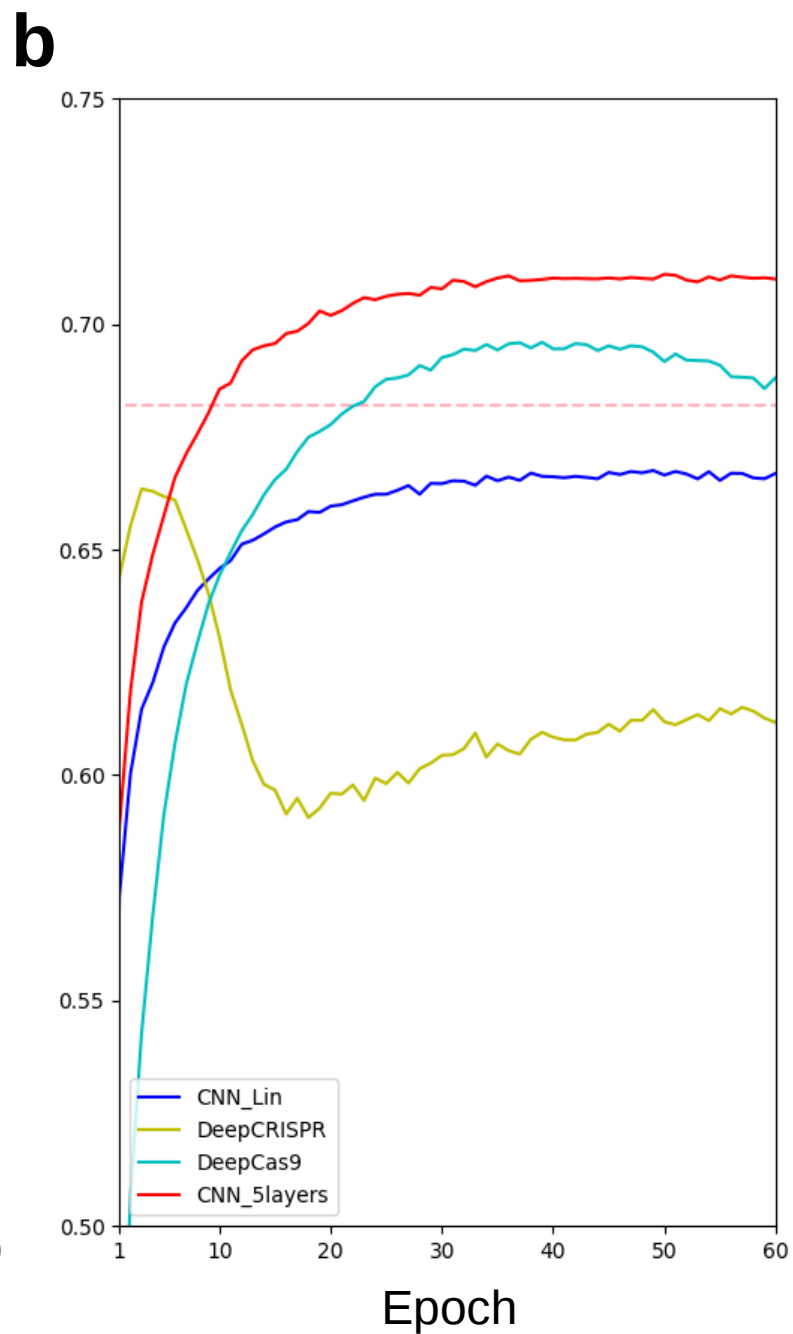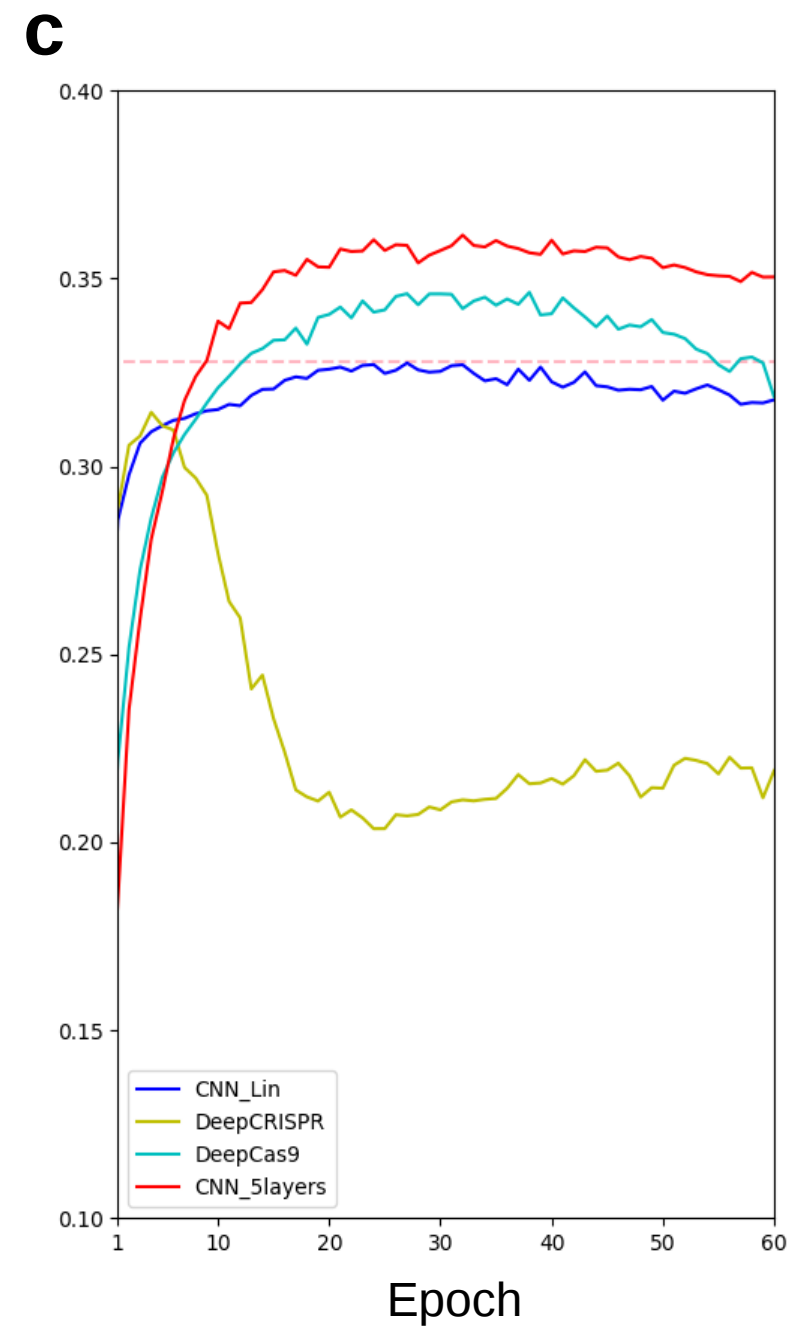

Supplement: Supplementary file 2 — Additional file 2 Figure S1. Real-time performance comparison under 5-fold cross-validation. The figure shows the real-time average Spearman correlation coefficients changes during being trained for several network architectures. Horizontal x-axis is training epochs, and vertical y-axis is average test Spearman correlation coefficients under 5-fold cross-validation. a, b and c represent Cas9, eSpCas9 and Cas9 (△recA), respectively. The light pink dashed are respectively corresponding to Spearman correlation coefficients of 0.542, 0.682 and 0.328 for Cas9, eSpCas9 and Cas9 (△recA), which are performances of gradient boosting regression trees. [file 12859_2019_3151_MOESM2_ESM.pdf]

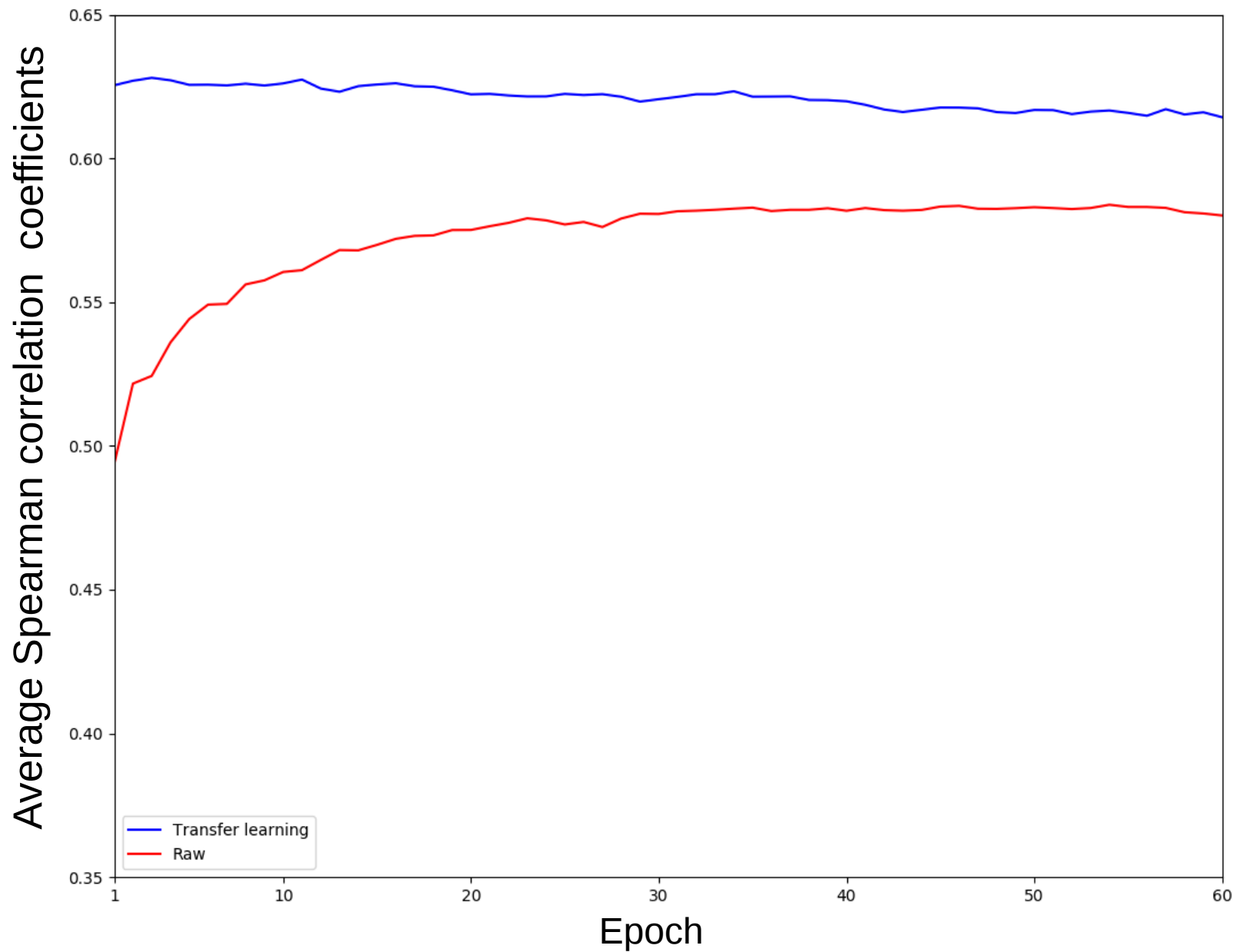

Supplement: Supplementary file 6 — Additional file 6 Figure S2. Real-time performance comparison between raw and transfer learning scenario. The figure shows the real-time average Spearman correlation coefficients changes during being trained in raw and transfer learning scenario. Horizontal x-axis is training epochs, and vertical y-axis is average test Spearman correlation coefficients under 5-fold cross-validation. [file 12859_2019_3151_MOESM6_ESM.pdf]
